# Comparing virus incubation time in SIRC models: Deterministic versus stochastic approaches

**DOI:** 10.1016/j.idm.2025.08.002

**Published:** 2025-08-09

**Authors:** Abdelmalik Moujahid, Fernando Vadillo

**Affiliations:** a Department of Computer Science and Technology, Universidad Internacional de la Rioja, Logroño, Spain; b Department of Mathematics, Universidad del País Vasco (UPV/EHU), Bilbao, Spain

**Keywords:** Epidemic dynamics, Delay differential equations, Stochastic delay differential equations

## Abstract

Time delays are a fundamental feature in modeling stochastic epidemic systems, as they capture the incubation period and other physiological lags inherent in disease transmission. In this work, we investigate a stochastic SIRC (Susceptible-Infectious-Recovered-Cross-immune) epidemic model where the delay is incorporated into the transmission term to reflect the incubation period. To account for environmental variability, we examine two stochastic formulations: the classical approach, which adds independent white noise to each compartment, and a probabilistic, event-driven model in which stochasticity arises directly from transition probabilities.

A key focus of our study is the comparison between different delay formulations in the transmission term, specifically contrasting the standard approach—where the delay acts only on the infected compartment—with alternative formulations that distribute the delay across both susceptible and infected populations. Through systematic numerical simulations, we find that the choice of delay formulation strongly influences the timing and magnitude of the initial epidemic peak, while the long-term (asymptotic) behavior is more robust but remains sensitive to the underlying stochastic framework. The probabilistic model, in particular, offers a more faithful depiction of correlated fluctuations and extinction phenomena, capturing the biological complexity of epidemic processes more accurately than the classical approach. These results underscore the importance of both the delay representation and the stochastic modeling strategy in shaping the qualitative and quantitative features of epidemic dynamics.

## 1 Introduction

The spread of infectious diseases is shaped by a complex interplay of time delays and randomness—two features that are fundamental to understanding epidemic dynamics. Delays arise naturally in epidemic systems, most notably through the incubation period: the time between when an individual is infected and when symptoms appear. During this latent phase, people may unknowingly transmit the disease to others, and the presence of asymptomatic carriers—who never develop symptoms but can still spread the virus—adds another layer of difficulty to containment efforts. Furthermore, differences in susceptibility, exposure, and intervention strategies across population groups introduce additional temporal complexity, as the pace and pattern of disease progression can vary widely within a community.

Equally important is the role of stochasticity, which captures the inherent randomness of disease transmission. Epidemics unfold in unpredictable ways, influenced by random events such as chance encounters, variations in individual behavior, and fluctuating environmental conditions. This randomness can have profound effects, leading to outcomes that diverge sharply from what deterministic models predict. For example, in small populations, a disease might die out by chance even when conditions seem ripe for an outbreak, or conversely, a rare sequence of events might trigger an unexpected epidemic wave.

In light of these complexities, mathematical models are an indispensable tool for understanding the complex dynamics of disease transmission within populations. The foundational literature in this field, epitomized by seminal works such as Murray's *mathematical biology* (Murray, 2002) and the comprehensive account by de Vries et al. (de et al., 2006), serve as cornerstones for understanding the mathematical foundations of epidemic phenomena. In addition, scientific contributions such as Chou's *Mathematical and Statistical Methods for Multi-state Health Outcomes* (Chou & Friedman, 2016) are invaluable insights into advanced modeling methods. The seminal works of Hethcote (Daley & Gani, 1999) and Castillo-Chavez (Hethcote, 2000, pp. 599–653) further enrich our understanding of epidemiological dynamics. Recent studies have emphasized the importance of comparing deterministic and stochastic epidemic models, particularly in the context of extinction times and the influence of model structure on disease persistence (Moujahid & Vadillo, 2021). Other works have addressed the challenge of parameter estimation in stochastic differential models, highlighting methods for calibrating such models using stochastic data and advanced optimization techniques (Moujahid & Vadillo, 2022). The role of time delays, especially in the context of stochastic systems, has also received increasing attention, as delays can fundamentally alter both transient and long-term epidemic dynamics (Moujahid & Vadillo, 2023).

For a more in-depth investigation, Castillo's *Mathematical Modeling of Biological Systems* (Martcheva, 2015) provides an exhaustive exploration of the intricacies of modeling, while Martcheva's monograph (Martcheva, 2015) and the collaborative efforts of Castillo et al. (Brauer et al., 2019) deal with sophisticated concepts that are essential for navigating complex epidemiological terrain.

However, real epidemics rarely follow average trajectories. Stochasticity—arising from random variations in contact patterns, individual responses, and environmental factors—can lead to pronounced fluctuations, unexpected extinctions, and complex transient dynamics that deterministic models cannot capture. To address this, researchers have developed a range of stochastic modeling approaches. Some introduce random noise directly into the equations, representing fluctuations in transmission rates or population sizes (Alsakaji et al., 2022; Liu & Jiang, 2020; Rajasekar & Pitchaimani, 2020; Rihan, 2021; Zhao & Jiang, 2014). Others build models from the ground up using probabilistic rules for transitions between disease states, leading to event-driven descriptions that more faithfully capture the discrete, random nature of real epidemics (Allen, 2007, 2010; Vadillo, 2019).

This study builds on these foundations by applying both classical and probabilistic stochastic modeling techniques to the SIRC (Susceptible-Infectious-Recovered-Cross-immune) epidemic framework. In this context, the variable *S*(*t*) represents the proportion of individuals in the population who are susceptible to infection at time *t*—that is, those who have not yet contracted the disease and remain at risk. Meanwhile, *I*(*t*) denotes the proportion of individuals who are infectious at time *t*, actively carrying and capable of transmitting the disease to others. A central focus is the effect of incorporating time delays into the transmission term, representing the biological reality of incubation periods. In particular, we consider two distinct approaches to formulating the delay in the transmission term: the original formulation, which uses the incidence term *S*(*t*)*I*(*t* − *τ*), and an alternative formulation that employs *S*(*t* − *τ*)*I*(*t* − *τ*). By systematically analyzing how these delays interact with different forms of stochasticity, this work aims to clarify their combined impact on epidemic behavior and to illuminate the mechanisms that drive disease spread in populations.

The structure of this paper reflects this approach. We begin by introducing the deterministic SIRC model and exploring its temporal dynamics in the presence of delays. We then present two stochastic extensions: one that incorporates Gaussian noise into the deterministic framework, and another that adopts a probabilistic, event-driven perspective. Finally, we analyze the results of numerical simulations and discuss their broader implications for epidemic modeling and public health.

## 2 The SIRC epidemic delay model

The seasonally forced SIRC model provides a robust framework to capture the complicated dynamics of influenza A transmission over time (Casagrandi et al., 2006). Influenza A exhibits distinct seasonal patterns, with infection peaks occurring during specific periods of the year. The seasonally forced SIRC model takes into account external factors, such as environmental conditions and human behavior, that influence the transmission of the virus. A key feature of the seasonally forced SIRC model is its ability to predict a variety of complex temporal patterns that closely resemble real-world observations of influenza A dynamics.

The population dynamics in the SIRC model are divided into four different compartments, each representing a specific category of individuals. These compartments include *S*(*t*), which represents the proportion of susceptible individuals; *I*(*t*), which denotes the proportion of individuals who are currently infected with the disease; *R*(*t*), which indicates the proportion of individuals who have recovered from the infection and acquired immunity; and *C*(*t*), which represents the proportion of individuals with cross-immunity due to previous exposure to related pathogens or vaccination.

In the deterministic formulation of epidemic models, it is typically assumed that environmental variability has a negligible effect on the transmission dynamics. As a result, the evolution of the epidemic can be described by a deterministic SIRC model in which delay is incorporated directly into the equations. The most common approach, and the one adopted in this work, introduces a fixed delay *τ* in the infection term, so that the incidence of new infections at time *t* depends on the current number of susceptibles and the number of infectives at a previous time, specifically through the term *S*(*t*)*I*(*t* − *τ*). This formulation is motivated by the biological reality of diseases such as influenza A, where the time between exposure and the onset of infectiousness—the incubation period—plays a crucial role in disease spread.

### 2.1 The original delay formulation

The dynamics of the SIRC model with time delay, according to our original formulation, are governed by the following system of ordinary differential equations:

(1) $$\left\{\begin{array}{l} \dot{S}\left(t\right)=\eta \left(1-S\left(t\right)\right)-\xi S\left(t\right)I\left(t-\tau \right)+\beta C\left(t\right),\;\\ \dot{I}\left(t\right)=\xi S\left(t\right)I\left(t-\tau \right)+\sigma \xi C\left(t\right)I\left(t\right)-\left(\eta +\alpha \right)I\left(t\right),\;\\ \dot{R}\left(t\right)=\left(1-\sigma \right)\xi C\left(t\right)I\left(t\right)+\alpha I\left(t\right)-\left(\eta +\gamma \right)R\left(t\right),\;\\ \dot{C}\left(t\right)=\gamma R\left(t\right)-\xi C\left(t\right)I\left(t\right)-\left(\eta +\beta \right)C\left(t\right),\; \end{array}\right)$$

where *τ* denotes the incubation period, and the parameters *η*, *β*, *ξ*, *σ*, *α*, and *γ* represent the mortality rate, the rate at which cross-immune individuals revert to the susceptible class, the transmission rate, the average reinfection probability for cross-immune individuals, the recovery rate, and the rate at which recovered individuals become cross-immune, respectively. The model is supplemented with initial conditions for all compartments on the interval *θ* ∈ [−*τ*, 0]:

$$\begin{array}{lll} S\left(\theta \right)=\phi_{1}\left(\theta \right),\;I\left(\theta \right)=\phi_{2}\left(\theta \right), & & \\ R\left(\theta \right)=\phi_{3}\left(\theta \right),\;C\left(\theta \right)=\phi_{4}\left(\theta \right). & & \end{array}$$

This formulation, which follows the methodology in (Rihan, 2021), is designed to reflect the biological incubation period characteristic of many respiratory infections. The model admits a disease-free equilibrium *ɛ*_0_ = (1, 0, 0, 0) and, when the basic reproduction number $R_{0}=\frac{\xi }{\eta +\alpha }>1$, a positive endemic equilibrium *ɛ*_+_ = (*S*∗, *I*∗, *R*∗, *C*∗). By explicitly incorporating the delay in the infection term, the model ensures that the force of infection at time *t* depends on those individuals who were infected at time (*t* − *τ*) and have just become infectious. This modeling choice, in which the incidence term is expressed as *S*(*t*)*I*(*t* − *τ*), more accurately reflects the biological reality that the current pool of susceptible is at risk from individuals who have recently completed their incubation period. This approach is particularly relevant in contexts where the susceptible population is dynamic, influenced by demographic processes and transitions between immunity states, as captured by the structure of the model.

### 2.2 The alternative delay formulation

It is important to recognize that alternative methods for incorporating delay into epidemic models have been explored in the literature (Goel et al., 2023). One such approach considers a delayed incidence term of the form *S*(*t* − *τ*)*I*(*t* − *τ*). In this formulation, the equation for the susceptible compartment uses the standard, non-delayed incidence term *S*(*t*)*I*(*t*), ensuring that susceptibles are depleted immediately upon exposure. In contrast, the infectious compartment is updated using the delayed incidence term *S*(*t* − *τ*)*I*(*t* − *τ*), so that new infectious individuals appear only after a fixed incubation period, *τ*, following exposure.

This structure introduces an asynchronous update between the two compartments: the reduction in susceptibles occurs at the moment of exposure, while the corresponding increase in the infectious population is postponed by the incubation period. As a result, the susceptible population responds instantly to new exposures, accurately reflecting real-time changes in risk or contact patterns. However, the emergence of new infectious cases is delayed, as only those individuals who were exposed *τ* units of time earlier become infectious at the current time. This temporal mismatch creates a lag between the depletion of susceptibles and the rise in infectious individuals, leading to a period during which individuals have left the susceptible pool but have not yet contributed to the infectious pool. Such a formulation captures the biological reality of incubation periods and can significantly influence the timing and shape of epidemic dynamics (Goel et al., 2023; Guglielmi et al., 2022).

The SIRC model equations under this alternative delay formulation can be written as:

(2) $$\left\{\begin{array}{l} \dot{S}\left(t\right)=\eta \left(1-S\left(t\right)\right)-\xi S\left(t\right)I\left(t\right)+\beta C\left(t\right),\;\\ \dot{I}\left(t\right)=\xi S\left(t-\tau \right)I\left(t-\tau \right)+\sigma \xi C\left(t\right)I\left(t\right)-\left(\eta +\alpha \right)I\left(t\right),\;\\ \dot{R}\left(t\right)=\left(1-\sigma \right)\xi C\left(t\right)I\left(t\right)+\alpha I\left(t\right)-\left(\eta +\gamma \right)R\left(t\right),\;\\ \dot{C}\left(t\right)=\gamma R\left(t\right)-\xi C\left(t\right)I\left(t\right)-\left(\eta +\beta \right)C\left(t\right).\; \end{array}\right)$$

The choice between these formulations can have significant implications for the predicted timing and magnitude of epidemic peaks, as well as for the overall qualitative behavior of the system.

### 2.3 Numerical comparison of delay formulations in the deterministic case

This section delves into the impact of two distinct delay formulations in the infection process of the deterministic SIRC (Susceptible-Infectious-Recovered-Cross-immune) model. The comparison centers on how the mathematical structure of the delay term—either acting solely on the infected compartment or on both susceptible and infected compartments—shapes the epidemic's temporal evolution.

**Fig. 1:**
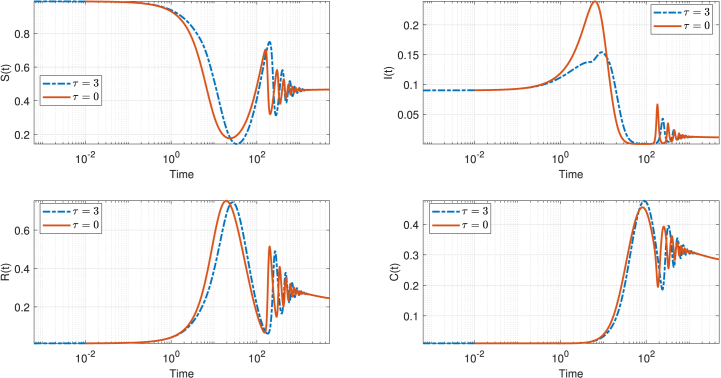
Comparison of the time evolution of the solutions of the deterministic SIRC model (1) for *τ* = 0 (no delay) and *τ* = 3, highlighting the impact of introducing a time delay on the asymptotic behavior. The extended time scale reveals that the delayed system (*τ* = 3) exhibits more pronounced oscillations that persist longer before converging to equilibrium, while the non-delayed system (*τ* = 0) approaches steady state more rapidly with dampened oscillations. The model parameters are set as follows: *η* = 0.0005, *ξ* = 0.6, *β* = 0.01, *σ* = 0.12, *α* = 0.3, and *γ* = 0.02, which leads to a calculated reproduction number of *R*_0_ = 1.9967.

Fig. 1 illustrates the asymptotic behavior of the SIRC model by comparing the temporal evolution of each compartment in the presence (*τ* = 3) and absence (*τ* = 0) of a time delay in the infection process. In contrast to the initial transient phase, the asymptotic regime reveals that the inclusion of a delay (*τ* = 3) leads to more persistent and higher-amplitude oscillations in all compartments compared to the no-delay case. Specifically, the trajectories for *S*(*t*), *I*(*t*), *R*(*t*), and *C*(*t*) in the delayed model (blue curves) exhibit sustained oscillatory behavior, with the amplitude of oscillations decaying more slowly over time. In comparison, the no-delay model (orange curves) demonstrates a more rapid attenuation of oscillations, with all compartments approaching their respective equilibrium values more smoothly.

This difference in asymptotic behavior highlights the significant impact of the incubation period on the long-term dynamics of the epidemic. Specifically, the presence of a delay introduces a memory effect into the system, resulting in more persistent and pronounced oscillatory dynamics across the population compartments. In contrast, when no delay is present, the system tends to stabilize more rapidly, with oscillations dampening at a faster rate. These findings underscore the importance of explicitly modeling the incubation period to accurately capture the full spectrum of epidemic outcomes, particularly the persistence of oscillations in the endemic regime.As the epidemic progresses, additional factors such as the development of natural immunity and the implementation of interventions further limit transmission, contributing to a decline in infection rates. The interplay between susceptible and infected individuals, combined with periodic fluctuations in transmission, can reinforce or attenuate oscillatory behavior in the infection curve. Ultimately, these oscillations diminish over time, and the system approaches a stable endemic level or, in some cases, disease eradication, reflecting the effectiveness of control measures and the natural course of immunity within the population.

**Fig. 2:**
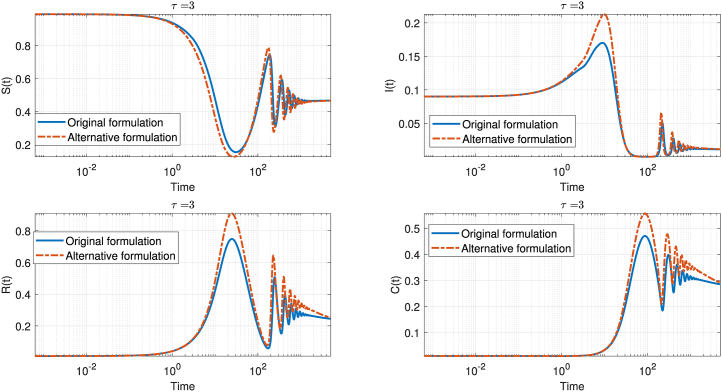
Comparison of the original and alternative delay formulations in the deterministic SIRC model for *τ* = 3. The four subplots show the time evolution of each compartment: susceptible (S), infected (I), recovered (R), and cumulative cases (C). Solid lines represent the original formulation, while dashed lines correspond to the reviewer's suggested alternative. The trajectories are plotted on a logarithmic time scale to emphasize both short- and long-term dynamics. The model parameters are as in Fig. 1.

Fig. 2 presents a comparative analysis of the original and alternative delay formulations in the SIRC model for *τ* = 3. In this figure, the original formulation is represented by the solid blue curve, while the alternative formulation, shown as a dashed orange curve. Each panel displays the time evolution of one compartment—susceptible *S*(*t*), infected *I*(*t*), recovered *R*(*t*), and cross-immune *C*(*t*) with the time axis on a logarithmic scale to emphasize the asymptotic regime.

The comparison reveals that the alternative formulation leads to oscillations of greater amplitude and longer persistence across all compartments. In particular, the peaks in the infected and recovered populations are higher and more sustained when the delay is incorporated into both the susceptible and infected compartments. This effect is also evident in the susceptible and cross-immune compartments, where the alternative approach results in larger deviations from equilibrium and a slower return to steady state. While both formulations eventually approach similar qualitative long-term behavior, the quantitative differences in oscillatory dynamics and equilibrium values are substantial. These findings highlight the importance of the specific mathematical representation of delay in epidemic models, as it can significantly influence both the transient and asymptotic dynamics of disease spread.

Examining the trajectories, it is evident that the alternative formulation produces oscillations of greater amplitude and with a noticeable phase shift compared to the original model. This difference is particularly pronounced in the susceptible and recovered compartments, where the alternative model consistently predicts larger deviations from the equilibrium state. In the infected and cross-immune compartments, the alternative formulation also leads to higher peaks and more persistent oscillations, indicating a slower decay toward the steady state.

Despite these differences in the transient and asymptotic dynamics, both formulations eventually approach similar qualitative behavior, with the oscillations gradually dampening and the system tending toward equilibrium. However, the alternative formulation results in slightly higher steady-state values for the susceptible and cross-immune populations, as well as a more prolonged approach to equilibrium.

Overall, the figure highlights that the mathematical representation of the delay in the incidence term has a substantial impact on both the amplitude and persistence of epidemic oscillations. By introducing the delay into both the susceptible and infected compartments, the alternative formulation enhances the memory effect within the system, resulting in more pronounced and long-lasting oscillatory dynamics. This underscores the importance of carefully selecting the delay structure in epidemic models, as it can significantly influence the predicted course and control of infectious diseases.

## 3 The classical stochastic delay SIRC model

From a biological perspective, it is crucial to include environmental variability in epidemic models to accurately represent the dynamics of disease transmission. A straightforward method is to introduce white noise terms proportional to the susceptible, infected, recovered, and cross-immune compartments (*S*(*t*), *I*(*t*), *R*(*t*), and *C*(*t*), respectively). This approach accounts for the stochastic nature of environmental factors influencing disease spread. The resulting stochastic delay differential equation (SDDE) model under the original delay formulation is given by:

(3) $$\left\{\begin{array}{l} dS\left(t\right)\;=\;\left[\eta \;\left(1-S\left(t\right)\right)-\xi \;S\left(t\right)\;I\left(t-\tau \right)+\beta \;C\left(t\right)\right]\;dt\;+\;\nu_{1}\;S\left(t\right)\;dW_{1}\left(t\right),\;\\ dI\left(t\right)\;=\;\left[\xi \;S\left(t\right)\;I\left(t-\tau \right)+\sigma \;\xi \;C\left(t\right)\;I\left(t\right)-\left(\eta +\alpha \right)\;I\left(t\right)\right]\;dt\;+\;\nu_{2}\;I\left(t\right)\;dW_{2}\left(t\right),\;\\ dR\left(t\right)\;=\;\left[\left(1-\sigma \right)\;\xi \;C\left(t\right)\;I\left(t\right)+\alpha \;I\left(t\right)-\left(\eta +\gamma \right)\;R\left(t\right)\right]\;dt\;+\;\nu_{3}\;R\left(t\right)\;dW_{3}\left(t\right),\;\\ dC\left(t\right)\;=\;\left[\gamma \;R\left(t\right)-\xi \;C\left(t\right)\;I\left(t\right)-\left(\eta +\beta \right)\;C\left(t\right)\right]\;dt\;+\;\nu_{4}\;C\left(t\right)\;dW_{4}\left(t\right).\; \end{array}\right)$$

where *W*_1_(*t*), *W*_2_(*t*), *W*_3_(*t*), and *W*_4_(*t*) denote four independent Brownian motions, and the positive constants *ν*_1_, *ν*_2_, *ν*_3_, and *ν*_4_ represent the intensity of these environmental white noises.

### 3.1 The stochastic SIRC model without delay

Before proceeding to the comparison between the two delay formulations discussed in the previous section—namely, the original formulation and the alternative formulation, where the delay acts on both the susceptible and infected compartment, we first examine the behavior of the stochastic SIRC model in the absence of delay. In this scenario, the model is governed by system (3) with *τ* = 0, so the infection process is instantaneous and does not incorporate any latent period between exposure and infectiousness. The simulation was performed using the parameter set *η* = 0.0005, *ξ* = 0.6, *β* = 0.01, *σ* = 0.12, *α* = 0.3, and *γ* = 0.02, corresponding to a basic reproduction number *R*_0_ = 1.9967. Environmental variability was introduced through white noise terms with intensities *ν*_1_ = 0.1 and *ν*_2_ = *ν*_3_ = *ν*_4_ = 0.02. The results shown are averages over 200 independent stochastic realizations, ensuring that the mean trajectories reflect the typical behavior of the system under random fluctuations.

**Fig. 3:**
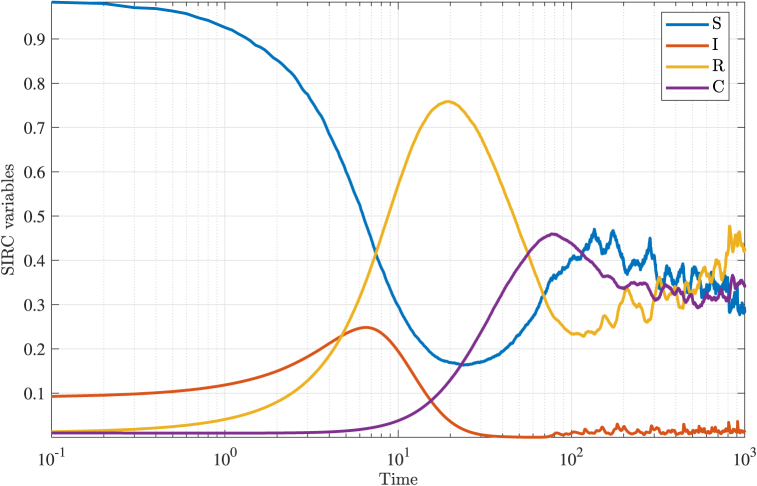
Evolution of the solution over time for the stochastic model (3) in the absence of delay. The model parameters are *η* = 0.0005, *ξ* = 0.6, *β* = 0.01, *σ* = 0.12, *α* = 0.3, *γ* = 0.02, which corresponds to a reproduction number *R*_0_ = 1.9967. The intensities of the white noise are *ν*_1_ = 0.1 and *ν*_2_ = *ν*_3_ = *ν*_4_ = 0.02. Stochastic trajectories were averaged over 1000 samples.

Fig. 3 displays the time evolution of the four compartments—susceptible *S*(*t*), infected *I*(*t*), recovered *R*(*t*), and cross-immune *C*(*t*)—for the stochastic SIRC model without delay. The trajectories reveal the characteristic progression of an epidemic: the susceptible population declines as individuals become infected, the infected compartment rises to a peak before decreasing as recovery and immunity accumulate, and the recovered and cross-immune compartments increase accordingly. Notably, the presence of stochasticity introduces visible fluctuations around the mean trajectories, particularly in the long-term regime, where the system does not settle to a fixed equilibrium but instead exhibits persistent random oscillations. These results highlight the impact of environmental noise on epidemic dynamics, even in the absence of delay, and provide a baseline for assessing the additional effects introduced by delayed infection processes.

A close examination of the long-term behavior reveals notable differences between the stochastic and deterministic SIRC models. In the stochastic setting, the proportion of susceptible individuals stabilizes at a lower value (around 0.3 %) than in the deterministic case (approximately 0.5 %), while the proportion of recovered individuals is correspondingly higher under stochastic dynamics. The average prevalence of infection remains similar in both models, indicating that noise does not substantially alter the mean level of infection. However, the deterministic model tends to overestimate the proportion of cross-immune individuals compared to its stochastic counterpart. These distinctions highlight how environmental variability can shift the equilibrium distribution of population compartments, particularly for the susceptible, recovered, and cross-immune groups, underscoring the importance of accounting for stochastic effects in epidemic modeling.

### 3.2 The stochastic SIRC model with delay

As in the previous deterministic analysis, we adopted the original formulation of the delay for the construction of our stochastic delay model. This choice is biologically motivated and consistent with standard approaches in the literature, where the delay represents the incubation period between exposure and infectiousness. To assess the impact of the delay formulation on the model's behavior, we also considered the alternative approach, where the delay acts on both the susceptible and infected compartments.

By simulating both stochastic models under identical conditions, we were able to compare their transient and asymptotic dynamics. This comparative analysis allows us to evaluate how the specific mathematical representation of the delay influences the amplitude and persistence of oscillatory behavior, as well as the long-term distribution of the compartments. Such investigations are essential, as stochastic delay differential equations (SDDEs) provide a more realistic framework for capturing the inherent randomness and memory effects present in real epidemic processes. The results, discussed in the following sections, highlight the importance of delay structure in shaping the qualitative and quantitative features of epidemic trajectories under environmental variability.

**Fig. 4:**
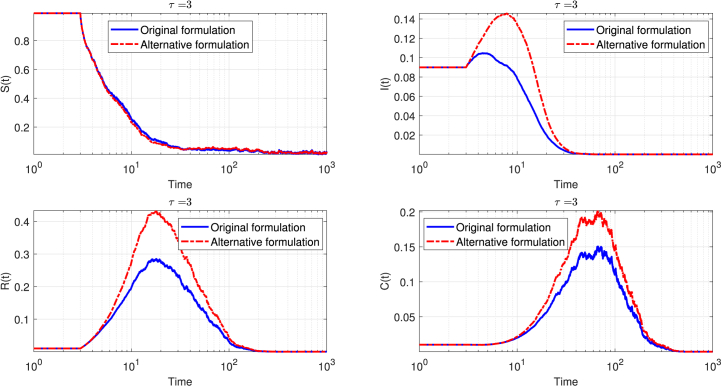
Comparison of the time evolution of the four compartments in the stochastic SIRC delay model for *τ* = 3, under the two different delay formulations: the original formulation (solid blue curve) and the alternative formulation (dashed red curve). Each panel displays the mean trajectory for one compartment—susceptible *S*(*t*), infected *I*(*t*), recovered *R*(*t*), and cross-immune *C*(*t*)—with the time axis on a logarithmic scale to highlight both transient and asymptotic regimes. The results demonstrate that the alternative formulation leads to oscillations of greater amplitude and longer persistence across all compartments, underscoring the significant impact of the delay structure on the stochastic epidemic dynamics.

Fig. 4 presents a comparative view of two delay formulations in a stochastic epidemic model (Eq. (3)) with a fixed delay parameter, *τ* = 3. Each subplot traces the temporal evolution of a key compartment: susceptible (*S*(*t*)), infected (*I*(*t*)), recovered (*R*(*t*)), and cross-immunity Compartment (*C*(*t*)), all plotted on logarithmic time scales to capture the full range of epidemic dynamics.

In the susceptible compartment, both formulations start with a high proportion of susceptibles. As the epidemic progresses, the number of susceptibles declines. The original delay formulation, represented by the solid blue curve, shows a slightly faster and more pronounced decrease, while the alternative delay, depicted by the dashed red curve, follows a similar but marginally slower trajectory. By the end of the simulation, both curves stabilize at low values, though the alternative delay leaves a slightly higher residual of susceptibles.

Turning to the infected compartment, both curves begin at low values. The alternative delay formulation produces a higher and broader peak, indicating a more intense and prolonged epidemic wave compared to the original delay. After reaching their respective peaks, both curves decline rapidly and converge toward zero, marking the resolution of the epidemic.

The recovered compartment rises as individuals transition out of the infectious state. Both delay formulations yield similar trajectories, with the alternative delay reaching a slightly higher maximum. The difference is most noticeable around the time of the epidemic peak, but both curves eventually plateau at comparable levels, reflecting the total number of individuals who have recovered.

In the cross-immunity compartment, both formulations again display similar patterns. The alternative delay produces a slightly higher and more prolonged peak, but the curves eventually decline and stabilize, indicating that the long-term burden of cross-immunity cases is comparable between the two approaches.

Overall, the figure demonstrates that while the alternative delay formulation accelerates the epidemic dynamics and amplifies the initial peaks, it does not lead to more persistent or pronounced oscillations in the long term. Both models ultimately display similar qualitative and quantitative behavior in the asymptotic regime, underscoring that the choice of delay structure primarily affects the timing and magnitude of the epidemic peaks rather than the long-term stability of the system.

### 3.3 Early outbreak dynamics: The role of the first infection peak

**Fig. 5:**
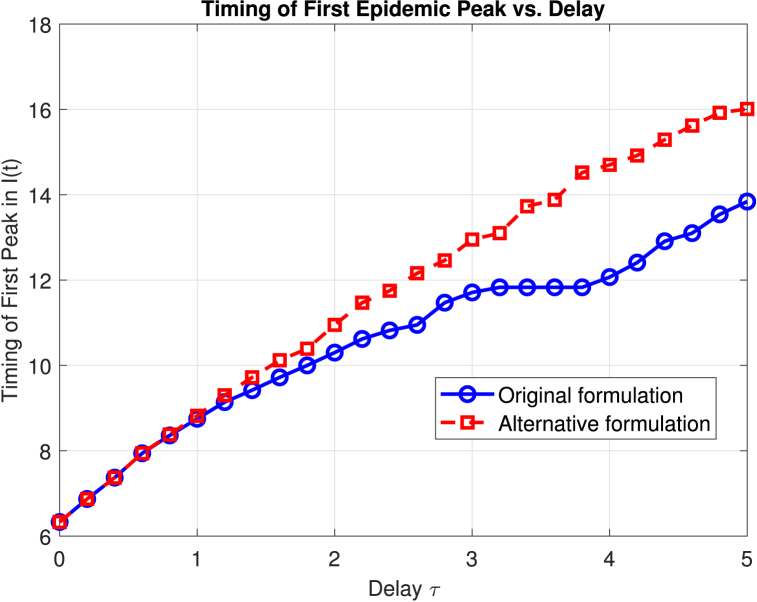
Timing of the first peak in the proportion of infected individuals, *I*(*t*), as a function of the delay parameter *τ* for the original (blue solid line with circles) and alternative (red dashed line with squares) stochastic SIRC model formulations. Increasing *τ* delays the epidemic peak in both models, with the alternative formulation consistently producing a later peak for each value of *τ*.

In addition to these observations, it is important to consider the epidemiological significance of the timing of the first peak in the proportion of infected individuals. This initial peak marks a critical juncture in the outbreak, reflecting the rapid transmission of infection through the susceptible population during the early phase of the epidemic. The timing and magnitude of this peak are influenced by factors such as the transmissibility of the pathogen, contact patterns within the population, and environmental conditions that may facilitate or hinder disease spread. Accurately capturing the onset and height of the first peak is essential for informing public health interventions, as it determines the window of opportunity for implementing control measures and allocating healthcare resources effectively.To systematically assess how the delay parameter *τ* and the choice of delay formulation affect the timing of the first epidemic peak, we conducted a series of simulations across a range of delay values for both the original (*S*(*t*)*I*(*t* − *τ*)) and alternative (*S*(*t* − *τ*)*I*(*t* − *τ*)) stochastic SIRC models. Fig. 5 summarizes the results, displaying the relationship between the delay parameter and the timing of the first peak in the infected compartment *I*(*t*) for each formulation.

As shown in Fig. 5, increasing the delay *τ* systematically postpones the occurrence of the first epidemic peak in both models. Notably, the alternative formulation consistently results in a later peak compared to the original formulation for the same value of *τ*, and this difference becomes more pronounced as the delay increases. These findings highlight that, while both models are sensitive to the incubation period, the specific mathematical structure of the delay term can substantially influence the speed at which the epidemic escalates. The alternative approach, by introducing a stronger memory effect, further delays the outbreak's critical phase, which may have important implications for the timing and effectiveness of intervention strategies.

## 4 A probabilistic stochastic SIRC model: matrix formulation

The probabilistic approach to stochastic epidemic modeling is based on the explicit enumeration of all possible transitions between compartments over a small time interval, as established in foundational works by Bartlett, Bailey, Greenwood, and Allen (Allen, 2017; Allen, 2008; Bailey, 1975; Bartlett, 1956; Greenwood et al., 2009). In this framework, the system state is represented by the vector

$$X\left(t\right)=\left[\begin{array}{l} S\left(t\right)\\ I\left(t\right)\\ R\left(t\right)\\ C\left(t\right) \end{array}\right],$$

where each component denotes the proportion or count of susceptible, infected, recovered, and cross-immune individuals, respectively.

For a sufficiently small interval Δ*t*, it is assumed that only one event can occur, and the SIRC model structure leads to exactly nine distinct transitions, each corresponding to a specific biological process such as infection, recovery, or loss of immunity. These transitions are summarized in Table 1, with each event defined by a change in the state vector and a transition probability proportional to its rate and the current state.

**Table 1:** Possible transitions in the SIRC model, their changes in the state vector, and corresponding transition probabilities for a small time interval Δ*t*.

Name	Change Δ**X**^(*j*)^	Probability per Δ*t*
Birth into susceptible	(1,0,0,0)^*T*^	*p*_1_ Δ*t* = *η*(1 − *S*(*t*))Δ*t*
Death of infected	(0,−1,0,0)^*T*^	*p*_2_ Δ*t* = *ηI*(*t*)Δ*t*
Death of recovered	(0,0,−1,0)^*T*^	*p*_3_ Δ*t* = *ηR*(*t*)Δ*t*
Death of cross-immune	(0,0,0,−1)^*T*^	*p*_4_ Δ*t* = *ηC*(*t*)Δ*t*
Loss of cross-immunity (C → S)	(1,0,0,−1)^*T*^	*p*_5_ Δ*t* = *βC*(*t*)Δ*t*
Infection (S → I, delayed)	(−1,1,0,0)^*T*^	*p*_6_ Δ*t* = *ξS*(*t*)*I*(*t* − *τ*)Δ*t*
Cross-immunity acquisition (C + I)	(0,*σ*,1 − *σ*,−1)^*T*^	*p*_7_ Δ*t* = *ξC*(*t*)*I*(*t*)Δ*t*
Recovery (I → R)	(0,−1,1,0)^*T*^	*p*_8_ Δ*t* = *αI*(*t*)Δ*t*
Loss of immunity (R → C)	(0,0,−1,1)^*T*^	*p*_9_ Δ*t* = *γR*(*t*)Δ*t*

### 4.1 Matrix formulation of drift and diffusion

Let *ν* be the 4 × 9 stoichiometry matrix whose *j*-th column is the change vector Δ**X**^(*j*)^ for event *j*, and let **p** be the 9 × 1 vector of event rates *p*_*j*_.

$$\nu =\left[\begin{array}{lllllllll} 1 & 0 & 0 & 0 & 1 & -1 & 0 & 0 & 0\\ 0 & -1 & 0 & 0 & 0 & 1 & \sigma & -1 & 0\\ 0 & 0 & -1 & 0 & 0 & 0 & 1-\sigma & 1 & -1\\ 0 & 0 & 0 & -1 & -1 & 0 & -1 & 0 & 1 \end{array}\right]$$

$$p=\left[\begin{array}{l} \eta \left(1-S\left(t\right)\right)\\ \eta I\left(t\right)\\ \eta R\left(t\right)\\ \eta C\left(t\right)\\ \beta C\left(t\right)\\ \xi S\left(t\right)I\left(t-\tau \right)\\ \xi C\left(t\right)I\left(t\right)\\ \alpha I\left(t\right)\\ \gamma R\left(t\right) \end{array}\right]$$

Drift (Mean Change):

(4) E(ΔX)=νpΔt

or, equivalently,

(5) $$\frac{dX}{dt}=\mu \left(X\left(t\right),X\left(t-\tau \right)\right)=\nu \;p$$

Diffusion (Covariance):

(6) $$E\left(\Delta X{\left(\Delta X\right)}^{T}\right)=\nu \;diag\left(p\right)\;\nu^{T}\;\Delta t$$

where diag(**p**) is the diagonal matrix with entries *p*_*j*_.

### 4.2 Stochastic delay differential equation (SDDE)

Taking the limit as Δ*t* → 0, the SIRC model is described by the following SDDE in matrix form:

(7) dX(t)=μ(t,X(t),X(t−τ))dt+B(t,X(t),X(t−τ))dW(t)

where:

• ***μ***(*t*, **X**(*t*), **X**(*t* − *τ*)) = *ν* **p** is the drift vector,
• **D** = *ν* diag(**p**) *ν*^*⊤*^ is the diffusion (covariance) matrix,
• **D**^1/2^ is any matrix square root of **D** (e.g., via Cholesky or eigen-decomposition),
• **W**(*t*) is a 4 × 1 vector of independent standard Brownian motions.

### 4.3 Remarks on delay formulation

In all simulations and analyses, the alternative delay formulation can be implemented by substituting *S*(*t* − *τ*)*I*(*t* − *τ*) for *S*(*t*)*I*(*t* − *τ*) in the infection transition probability *p*_6_ and in the corresponding drift and diffusion terms. This matrix-based probabilistic formulation ensures that the stochastic SIRC model accurately reflects both the discrete event structure and the delay mechanisms relevant for comparative study.

### 4.4 On the construction and validity of the diffusion approximation

By explicitly enumerating all possible transitions and deriving the corresponding probabilities from the model parameters, the probabilistic approach provides a principled and biologically faithful representation of epidemic dynamics. This methodology not only captures the correct scaling and correlations of fluctuations but also allows for a more accurate analysis of rare events such as extinction or fade-out, which are often overlooked in models that simply add white noise to deterministic equations. The influence of this approach is evident in the modern theory of stochastic epidemic models, where it serves as the foundation for both analytical results and numerical simulations.

The formulation of the stochastic SIRC model in this section is grounded in the well-established methodology of constructing diffusion approximations for continuous-time Markov processes, as detailed in foundational works by Bartlett, Bailey, Greenwood, and Allen (Allen, 2017; Allen, 2008; Bailey, 1975; Bartlett, 1956; Greenwood et al., 2009). In this framework, the drift and diffusion terms are derived directly from the enumeration of all possible transitions and their associated rates, ensuring that the stochastic model faithfully represents the underlying biological events.

### 4.5 Simulation of the probability stochastic model

**Fig. 6:**
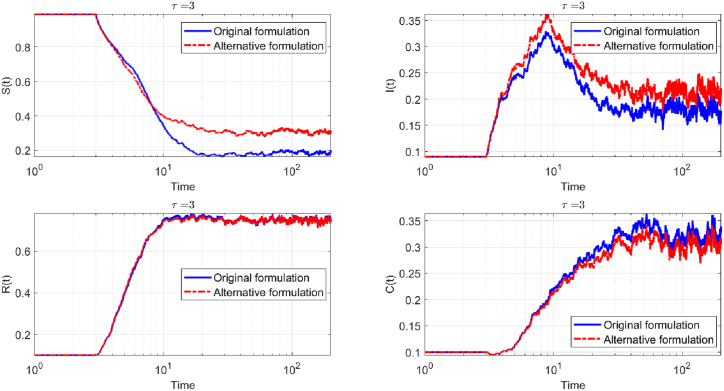
Time evolution of the four compartments in the stochastic SIRC model ?? under two different delay formulations: the original (blue solid curve), and the alternative formulatio (dashed red curve) for *τ* = 3. Parameter values are *η* = 0.0005, *ξ* = 0.6, *β* = 0.01, *σ* = 0.12, *α* = 0.3, *γ* = 0.02. Each trajectory represents the average over 200 stochastic realizations.

The numerical solution of the stochastic system ?? for various configurations of the delay parameter *τ* is presented in Fig. 6. The simulations were performed using the same parameter values as in previous analyses: *η* = 0.0005, *ξ* = 0.6, *β* = 0.01, *σ* = 0.12, *α* = 0.3, and *γ* = 0.02. To enhance statistical reliability and reduce the influence of random fluctuations, the results shown in the figure represent averages over a substantially larger number of independent stochastic realizations.

In the probabilistic, event-driven SIRC model, the epidemic trajectory is notably shaped by the choice of delay formulation. When the original delay is used (solid blue curves in Fig. 6), the susceptible population declines rapidly, resulting in a swift reduction of those at risk. The infectious compartment reaches a lower, sharper peak, indicating a concentrated outbreak that resolves quickly.

With the alternative delay formulation (dashed red curves), the decline in susceptibles is more gradual, leading to a higher final number of individuals who remain uninfected. The infectious compartment displays a broader and higher peak, reflecting a more prolonged and intense epidemic wave. Despite these differences, the recovered and cross-immunity compartments follow nearly identical paths for both delay formulations, stabilizing at similar values. This demonstrates that while the delay formulation significantly affects the timing and intensity of the epidemic, it has minimal impact on the eventual number of recoveries and cross-immunity cases.

Comparing the probabilistic, event-driven model to the classical stochastic SIRC model (see Fig. 4), it becomes apparent that the probabilistic approach is more robust to changes in delay formulations. In the classical model, switching between delay formulations leads to pronounced differences in the epidemic curves, particularly in the timing and magnitude of the peaks for the susceptible and infectious compartments. The classical model exhibits greater sensitivity, with more pronounced fluctuations and variability in outcomes depending on the delay structure.

In contrast, the probabilistic model maintains more consistent long-term outcomes across both delay formulations (see Fig. 6). The differences between the original and alternative delays are less dramatic, especially in the recovered and cross-immunity compartments, which converge to similar values regardless of the delay choice. This suggests that the probabilistic, event-driven model provides a more stable and reliable framework for epidemic prediction, even when the precise nature of the delay mechanism is uncertain. The model's intrinsic structure, grounded in event probabilities, appears to buffer the system against the variability introduced by different delay formulations, enhancing its robustness in practical applications.

**Fig. 7:**
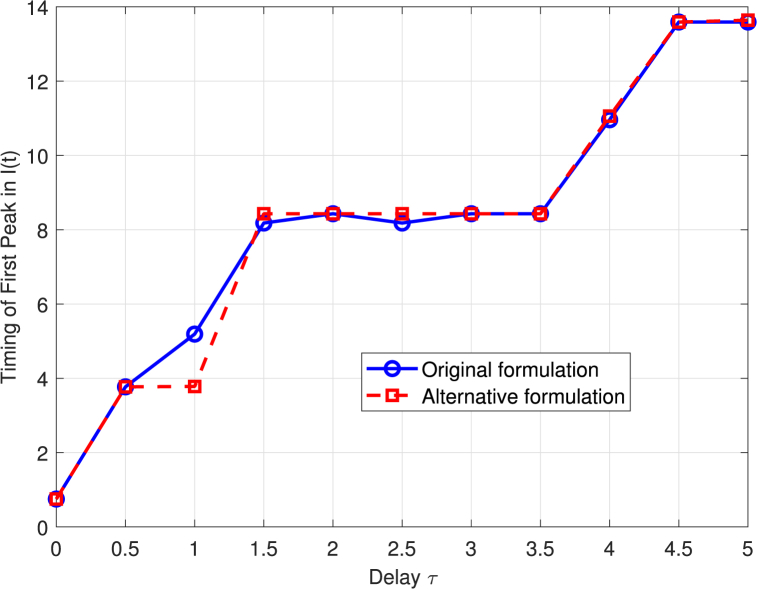
Timing of the first peak in the proportion of infected individuals, *I*(*t*), as a function of the delay parameter *τ* for the original (blue solid line with circles) and alternative (red dashed line with squares) stochastic probabilistic SIRC model formulations. For both formulations, increasing the delay parameter *τ* results in a later occurrence of the first peak in *I*(*t*).

Fig. 7 shows how the timing of the first peak in the proportion of infected individuals, *I*(*t*), varies with the delay parameter *τ* for two stochastic SIRC model formulations. The original formulation (blue solid line with circles) always produces an earlier peak than the alternative formulation (red dashed line with squares) for each value of *τ*.

Both formulations demonstrate that increasing the delay parameter, *τ*, systematically postpones the timing of the first peak in the proportion of infected individuals, *I*(*t*). The relationship between delay and peak timing is similar for both models, with the timing of the peak increasing in a stepwise manner as *τ* grows.

For practical purposes, this means that the specific choice between the original and alternative delay formulations does not substantially alter the qualitative or quantitative predictions regarding when the epidemic peak will occur. As *τ* becomes large, any minor differences between the two approaches diminish further, and their predictions converge. Thus, either formulation can be used to capture the essential effect of delay on epidemic timing in stochastic SIRC models, without significant impact on the main epidemiological conclusions.

## 5 Discussion

In this work, we have systematically compared two distinct stochastic models within the SIRC epidemic framework, each analyzed under two formulations for incorporating delay effects in the infection process. The first model (Eq. (3)) is the classical stochastic SIRC system, where independent white noise terms are added to each compartment to represent random fluctuations. The second (Eq. ??) is a probabilistic, event-driven model, in which both drift and diffusion terms are derived directly from the transition probabilities of the underlying epidemic events.

For both modeling approaches, we considered two biologically motivated ways to incorporate delay into the infection process:

• **Original delay formulation:** In this approach, the delayed incidence term *S*(*t*)*I*(*t* − *τ*) appears in both the susceptible and infectious equations. Specifically, susceptibles are depleted and new infectious cases arise according to the current number of susceptibles and the number of individuals who became infectious exactly *τ* units earlier. This formulation is widely used for diseases with a fixed incubation period, as it directly links new infections to the biological delay between exposure and infectiousness.
• **Alternative delay formulation:** Here, the incidence term takes the form *S*(*t* − *τ*)*I*(*t* − *τ*) and is introduced only in the equation for the infectious compartment. In this case, new infectious individuals appear after a delay, based on the state of both susceptibles and infectious individuals at time *t* − *τ*, while the susceptible compartment is updated without a delayed incidence term. This approach is appropriate when the depletion of susceptibles is assumed to occur immediately upon exposure, but the onset of infectiousness is delayed.

By systematically comparing these two delay structures within each stochastic modeling framework, we are able to disentangle the effects of different sources of stochasticity and to assess the epidemiological consequences of alternative assumptions about the timing of infection events. This comprehensive analysis clarifies how both the source of randomness and the structure of delay influence epidemic dynamics.

The classical stochastic SIRC model displays strong sensitivity to the choice of delay formulation, with pronounced differences in epidemic curves—particularly in the timing and magnitude of peaks in the susceptible and infectious compartments—when switching between delay structures. This model also exhibits greater variability and more pronounced fluctuations, as the externally imposed noise is not explicitly correlated between compartments.

In contrast, the probabilistic, event-driven model demonstrates greater robustness to changes in delay formulation. Long-term outcomes, especially in the recovered and cross-immunity compartments, remain consistent across different delay structures, with differences between original and alternative delays being less dramatic. The intrinsic event-driven nature of this model naturally incorporates correlations between compartments, resulting in smoother, more biologically plausible epidemic trajectories. As a result, the probabilistic model offers a more stable and reliable framework for epidemic prediction, even when the precise delay mechanism is uncertain, and is less affected by the variability introduced by alternative delay formulations.

As both models progress toward their asymptotic regimes, the differences in their long-term behavior become more apparent. The classical model typically reaches its permanent regime more quickly, with the amplitude of stochastic oscillations determined by the chosen noise intensities. The probabilistic model, on the other hand, exhibits a more gradual approach to equilibrium, with persistent but bounded oscillations that reflect the true variability of the underlying epidemic events. The amplitude and structure of these fluctuations scale naturally with event rates and population size, and correlations between compartments are preserved.

While both models ultimately display similar qualitative long-term behavior—settling into endemic states with ongoing random fluctuations—the probabilistic approach provides a more principled and accurate representation of the intrinsic variability in epidemic systems. This comparison highlights the importance of model choice when interpreting the stochastic dynamics of infectious disease outbreaks, particularly when precise characterization of variability and correlations is essential.

## 6 Conclusion

This study systematically compared deterministic and two distinct stochastic SIRC epidemic models, focusing on how different formulations of the incubation delay affect epidemic dynamics. The main finding is that the mathematical representation of the delay in the infection process—whether applied only to the infected compartment or to both susceptible and infected compartments—significantly influences the timing and magnitude of the initial epidemic peak. The alternative delay formulation, which introduces a stronger memory effect by delaying both compartments, tends to produce a later and more intense initial outbreak.

When comparing stochastic modeling approaches, the classical model (with independent white noise) is more sensitive to the choice of delay structure, resulting in greater variability in epidemic trajectories. In contrast, the probabilistic, event-driven model, which derives stochasticity from transition probabilities, demonstrates greater robustness: long-term outcomes, especially in the recovered and cross-immune compartments, remain consistent regardless of the delay formulation.

Overall, while both stochastic models eventually converge to similar endemic regimes characterized by persistent random fluctuations, the probabilistic approach provides a more biologically faithful and stable depiction of epidemic variability. These results underscore the importance of carefully selecting both the delay structure and the stochastic modeling framework when simulating infectious disease dynamics, as these choices can substantially impact predictions of outbreak timing, peak intensity, and the effectiveness of intervention strategies.

## CRediT authorship contribution statement

**Abdelmalik Moujahid:** Investigation. **Fernando Vadillo:** Investigation.

## Data availability statement

The numerical methods were implemented in matlab, the codes are available on request. The experiments were carried out in an Intel(R) Core(TM)i7-8665U CPU @ 1.90G.
